# Usefulness of Serum Carcinoembryonic Antigen (CEA) in evaluating response to chemotherapy in patients with advanced non small-cell lung cancer: a prospective cohort study

**DOI:** 10.1186/1471-2407-13-254

**Published:** 2013-05-22

**Authors:** Oscar Arrieta, Cynthia Villarreal-Garza, Luis Martínez-Barrera, Marcelino Morales, Yuzmiren Dorantes-Gallareta, Omar Peña-Curiel, Susana Contreras-Reyes, Eleazar Omar Macedo-Pérez, Jorge Alatorre-Alexander

**Affiliations:** 1 Department of Medical Oncology, National Cancer Institute, Mexico City, Mexico; 2 Laboratory of Experimental Oncology, National Cancer Institute, Mexico City, Mexico; 3 Universidad Nacional Autónoma de México, Mexico City, Mexico; 4 Department of Thoracic Oncology, National Institute of Respiratory Diseases, Mexico City, Mexico

**Keywords:** Carcinoembryonic antigen, Non small-cell lung cancer, Tumor markers, Prognosis, Response prediction

## Abstract

**Background:**

High serum carcinoembryonic antigen (CEA) levels are an independent prognostic factor for recurrence and survival in patients with non-small cell lung cancer (NSCLC). Its role as a predictive marker of treatment response has not been widely characterized.

**Methods:**

180 patients with advanced NSCLC (stage IIIB or Stage IV), who had an elevated CEA serum level (>10 ng/ml) at baseline and who had no more than one previous chemotherapy regimen, were included. CEA levels were measured after two treatment cycles of platinum based chemotherapy (93%) or a tyrosine kinase inhibitor (7%). We assessed the change in serum CEA levels and the association with response measured by RECIST criteria.

**Results:**

After two chemotherapy cycles, the patients who achieved an objective response (OR, 28.3%) had a reduction of CEA levels of 55.6% (95% CI 64.3-46.8) compared to its basal level, with an area under the ROC curve (AURC) of 0.945 (95% CI 0.91-0.99), and a sensitivity and specificity of 90.2 and 89.9%, respectively, for a CEA reduction of ≥14%. Patients that achieved a decrease in CEA levels ≥14% presented an overall response in 78% of cases, stable disease in 20.3% and progression in 1.7%, while patients that did not attain a reduction ≥14% had an overall response of 4.1%, stable disease of 63.6% and progression of 32.2% (p < 0.001). Patients with stable (49.4%) and progressive disease (22.2%) had an increase of CEA levels of 9.4% (95% CI 1.5-17.3) and 87.5% (95% CI 60.9-114) from baseline, respectively (p < 0.001). The AURC for progressive disease was 0.911 (95% CI 0.86-0.961), with sensitivity and specificity of 85 and 15%, respectively, for a CEA increase of ≥18%. PFS was longer in patients with a ≥14% reduction in CEA (8.7 vs. 5.1 months, p < 0.001). Reduction of CEA was not predictive of OS.

**Conclusions:**

A CEA level reduction is a sensitive and specific marker of OR, as well as a sensitive indicator for progression to chemotherapy in patients with advanced NSCLC who had an elevated CEA at baseline and had received no more than one chemotherapy regimen. A 14% decrease in CEA levels is associated with a longer PFS.

## Background

Lung cancer is the most common cause of cancer-related death in men and the second in women worldwide. It is responsible for approximately 1.4 million deaths per year [1]. Late diagnosis is common; more than 60% of patients present with stage IIIB/IV disease [2]. In addition, more than half of the remaining individuals treated with curative intent will experience relapse, and eventually succumb to their disease. The efficacy of chemotherapy (CT) in advanced disease is limited; with responses ranging from 20 to 35%; and a 1-year survival rate of 35% [3,4]. Virtually, all patients who initially respond will eventually progress.

Imaging studies remain the most objective available tool to evaluate response to CT, and a response to CT is a surrogate marker of clinical benefit, associated with a better survival outcome [5]. In addition, several measurements have been associated to response, such as changes or reduction of 18-fluorodeoxyglucose (FDG) metabolism evaluated by positron emission tomography (PET). Nevertheless, not all non-small-cell lung cancer (NSCLC) patients have measurable disease; thus complicating the possibility of evaluating objective responses. The value of serum markers will always be especially useful for cases where the clinical picture does not match the topographic measurements.

Regarding the use of markers as predictors of response to treatment in several types of malignant tumors, there are some antigens that have been proved useful. For example, in advanced prostate and ovarian cancer, the roles of prostate-specific antigen (PSA) and CA125, respectively, in predicting response to treatment and survival outcome have been clearly established and these markers are used routinely in clinical practice to monitor the effects of therapy [6].

The carcinoembryonic antigen (CEA) is an important marker for malignant tumors, including NSCLC. High serum CEA levels have been identified as a prognostic factor in both resected NSCLC [7-14] and metastatic disease [15,16]. However, the role of CEA as a predictive marker of response to CT has not been widely evaluated.

The objective of this study was to assess in a prospective manner both the sensitivity and the specificity of the changes in CEA levels and their relationship to response to CT treatment; as well as their association to progression free survival (PFS) and overall survival (OS) in patients with NSCLC.

## Methods

### Study population

Approval for this study was obtained from the Institutional Ethics Committee (010/059/ICI)(CB/675). Patients with the diagnosis of NSCLC treated at the National Cancer Institute of Mexico were recruited between February 2009 and May 2010. Inclusion criteria comprised: histologically proven diagnosis of NSCLC, patients with unresectable or metastatic disease, measurable disease, Eastern Cooperative Oncology Group (ECOG) performance status 0–2, life expectancy > 3 months, and patients cadidates for palliative first or second line CT. Patients with personal history of previous malignant neoplasms were excluded.

Tumor assessment by computed tomography was made at baseline and after two chemotherapy cycles. Only patients with CEA baseline levels > 10 ng/mL were included. Treatment consisted of a doublet platinum-based chemotherapy scheme or a tyrosine-kinase inhibitor (TKI). Initial response was determined by tomography using the established RECIST criteria [17].

Demographic data, medical history, and physical examination were performed before study entry. Height, weight, vital signs, ECOG performance status, and vital signs were assessed at every medical visit. CEA levels were measured at study entry before starting CT treatment and at the time of tomographic evaluation. Patients were followed until progression, death or last medical visit.

### CEA determination and analysis

Peripheral blood samples were obtained on day 1 before CT and after two CT cycles. Measurement was performed at the Clinical Pathology Laboratory of the National Cancer Institute of Mexico using a sequential chemoluminiscent immunoassay (Immulite 2000).

### Statistical analysis

With a descriptive purpose, we resumed continuous variables as arithmetic means, medians and standard deviations and categorical variables as proportions with 95% confidence intervals (95% CI’s). Sensitivity and specificity were calculated for the CEA levels and response measured by tomography. The association between CEA levels with overall response was calculated with Xi square test. Receiver operating characteristics (ROC) curve analysis to determine the best cut-off value for CEA levels to achieve a 90% specificity was undertaken. PFS was defined as the time-period from date of beginning of treatment to date of progressive disease by confirmed image or last follow-up, and OS was defined as the time-period from histological diagnosis to date of death or last follow-up visit. Survival was analyzed with the Kaplan-Meier method, and subgroups were compared with the log-rank and Breslow test. Statistical significance was determined with a p ≤ 0.05 in a two-sided test.

## Results

Between February 2009 and May 2010, a total of 426 patients with the diagnosis of advanced NSCLC were screened for CEA levels before the start of CT. One-hundred eighty patients (42%) with an abnormal baseline CEA level (>10 ng/mL) were prospectively recruited with a mean baseline CEA of 242.8 ng/mL (range, 10–7,440 ng/mL). Fifty-four percent were men and 46% were women (mean age, 59.4 ± 12.2 years). One hundred and three were smokers (57%) and seventy-three (41%) had wood-smoke exposure. Regarding histology, adenocarcinoma was the most common, being present in 152 patients (84%), as previously shown in our previous report [16]. In our study, CEA levels were lower in the non-adenocarcinoma histology group subtype compared to the adenocarcinoma, although this was not significantly different (128.9 vs. 264.3 ng/dl, p = 0.881). From the 180 patients with elevated CEA levels, 93.3% (168 patients) received a platinum-based chemotherapy, while 6.7% (12 patients) received a TKI (Table 1).

**Table 1:** Baseline patient and tumor characteristics

**N = 180**		**Mean ± SD**	**Patients**	**n (%)**
**Age (years)**		59.4 ± 12.2		
**Gender**	Women		82	(45.6)
	Men		98	(54.4)
**Smoking History**	Positive		103	(57.3)
	Negative		77	(42.7)
**Wood-smoke exposure**	Positive		73	(40.6)
	Negative		103	(59.4)
**ECOG**	0		38	21.1)
	1		115	(63.9)
	2		26	(14.4)
	3		1	(0.6)
**Clinical Stage**	III		28	(15.6)
	IV		152	(84.4)
**Histology**	Adenocarcinoma		152	(84.4)
	Squamous-cell		14	(7.8)
	Undifferentiated		13	(7.2)
	Large-cell carcinoma		1	(0.6)
**Baseline CEA**		242.8 ± 685		
**Treatment**	Platinum-Based CT		168	(93.3)
	Tyrosine-kinase			
	inhibitors		12	(6.7)
**Tumor ResponseEvaluation**	Complete/Partial		51	(28.3)
	Stable Disease		89	(49.4)
	Progressive Disease		40	(22.2)

Abbreviations: *SD*, standard deviation; *CEA*, carcinoembryonic antigen; *CT*, chemotherapy.

Objective response (complete plus partial response, OR), stable disease (SD) and progressive disease (PD) were 28.3, 49.4 and 22.2% respectively. Patients with OR had a CEA level reduction of 55.6% (95% CI 64.3-46.8); while patients with SD and PD had an increase of 9.4% (95% CI 1.5 to 17.3) and 87.5% (95% CI 60.9 to 114), respectively (p < 0.001).

**Figure 1:**
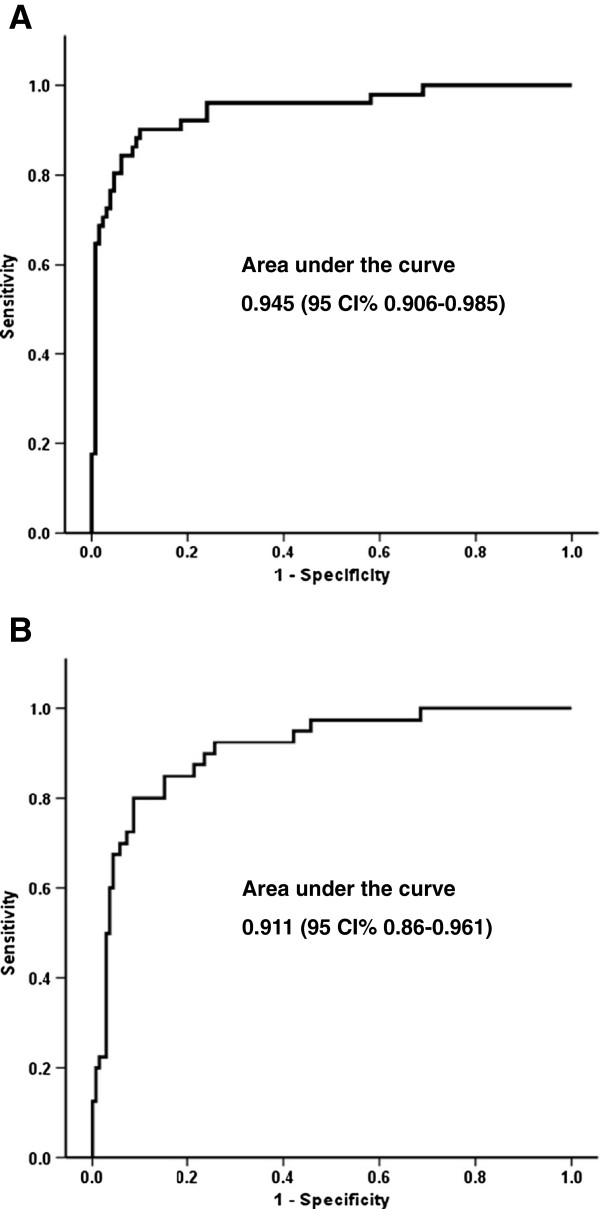
**Correlation between CAE levels and response. A**. ROC curve for CEA levels and overall response. **B**. ROC curve for CEA levels and progressive disease.

The ROC curve analysis for the changes in CEA levels in responsive patients had an area under the curve (AUC) of 0.945 (95% CI 0.91 to 0.99; Figure 1A). Sensitivity and specificity were of 90.2 and 89.9%, respectively for a CEA level reduction of 14% or greater. Patients that achieved a decrease in CEA levels ≥14% presented an overall response in 78% of cases, stable disease in 20.3% and progression in 1.7%, while patients that did not attain a reduction ≥14% had an overall response of 4.1%, stable disease of 63.6% and progression of 32.2% (p < 0.001). When we analyzed the CEA level decline associated with tumor response specifically in patients with non-adenocarcinoma, we found that patients with a reduction of ≥14%, had an overall response of 66.7%, stable disease 16.7%, and progression 16.7%, compared to the non-adenocarcinoma patients who did not achieved a reduction of ≥14% with a tumor response of 9.1%, 59.1% and 31.8%, respectively (p = 0.009).

Although the number of evaluated patients treated with EGFR-tyrosine kinase was limited (12 patients), patients with reduction in CEA levels ≥14%, had an overall response of 100%, compared to patients that did not have a CEA level reduction who achieved an overall response in 0%, stable disease in 70%, and progressive disease in 30% (p = 0.002).

The AUC in progressive disease was 0.911 (95% CI 0.86 to 0.96; Figure 1B), with a sensitivity and specificity of 85 and 15%, respectively, for a CEA level increase of 18% from baseline.

**Figure 2:**
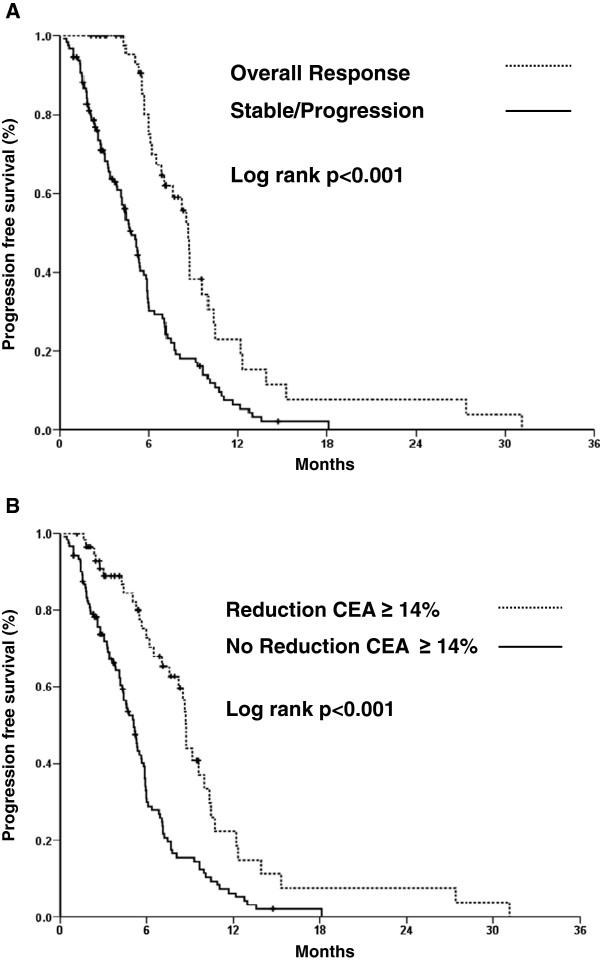
**Progression free survival in patients who have radiological response and CEA level reduction. A**. Kaplan-Meier curve comparing PFS in overall response vs stable/progressive disease. **B**. Kaplan-Meier curve comparing PFS in patients with a ≥ 14% reduction of CEA levels.

Median follow-up time was of 11.8 ± 8.4 months. According to RECIST criteria, patients that achieved OR had a superior PFS compared to patients with stable or progressive disease (Figure 2A). Similarly, PFS was longer in patients with a ≥14% reduction in CEA (8.7 months [CI 95% 8.4 to 9.0] vs. 5.1 months [CI 95% 4.5 to 5.8], p < 0.001; Figure 2B). Neither reduction of CEA (p = 0.48) nor OR measured by RECIST (p = 0.28) were predictive of OS.

## Discussion

CEA is a glycoprotein product of the gene *CEACAM-*5. It is a member of the immunoglobulin super family that serves as a cell-adhesion molecule and may also play a role in innate immunity [18]. CEA is often overexpressed in many malignant neoplasms including NSCLC and is readily detected in serum samples making it a valuable tool for the follow-up and prognosis of patients.

The role of CEA as a prognostic factor has been well established in colon cancer and is now part of the routine follow-up evaluation recommended by the current NCCN guidelines [19-22]. Moreover, Iwanicki et al. showed that the CEA kinetic allowed accurate evaluation of progression, response, and PFS in metastatic colon cancer suggesting an important role in objective response assessment [23]. In NSCLC, many studies evaluating CEA and prognosis have been written with contrasting results in the perioperative setting, some showing its role as a prognostic value [8,10,13,24] and others not confirming it [25-27].

The method for assessing treatment response in cancer patients is through the change in tumor size measured by computed tomography [17]. Though objective and well validated, it has inadequacies in daily practice settings as in the case of patients with pleural effusions, diffuse nodules, or tumors with poorly defined margins. Erasmus et al [28]. demonstrated that these measurements are often inconsistent and can lead to incorrect interpretation of tumor response; thus mandating for novel strategies in response evaluation. It would be especially useful to have a serum marker that can correlate with response in this particular setting. In addition, for advanced patients who are submitted to multiple imaging studies for follow-up and monitoring of progression, the assessment of a particular serum marker can obviate more time-consuming and expensive imaging evaluations, and can guide the clinician on the timing to request further studies when its elevation suggests progression. In this regard, five recent studies have reported that in NSCLC patients, the CEA levels can correlate with response to treatment.

A recent retrospective report by Ishiguro et al. [29] of 24 Japanese patients with resectable NSCLC showed a significant decrease of serum CEA levels after neoadjuvant chemotherapy in patients achieving partial response. They found a 60% reduction of CEA levels as an appropriate cutoff value for good response by ROC curve analysis; at this set-point, they found a sensitivity of 82.8% and a specificity of 69.2% for achieving objective response.

The prognostic and predictive value of pretreatment serum levels of CEA have been assessed in advanced NSCLC patients exclusively treated with gefitinib and erlotinib and, owing that conflicting results have been reported a direct relationship between high levels of CEA and response to EGFR-TKI, however its utility has not yet been established. Chiu et al. [30] and Xu et al. [31] assessed the clinical value of CEA in prediction of EGFR-TKI therapy response in advanced NSCLC patients. The former authors found an association between image response and tumor marker assessment at 4 weeks of therapy. Unexpectedly, they found no association between CEA and PFS nor OS. The latter authors assessed CEA levels at baseline and after 4 weeks of an EGRF-TKI in advanced NSCLC patients; they found that a decrease of ≥32% from baseline was closely related to OR and a longer median survival time, which confirms our findings. In the other hand, Okamoto et al [32] and Jung et al [33] reported that patients treated with EGFR-TKI with high pretreatment levels of CEA had a longer survival and a better response than those with low CEA levels.

Ardizzoni et al. [15] explored the value of CEA in advanced NSCLC patients receiving platinum-based CT. They found that a reduction of ≥20% of CEA after 2 cycles of CT had accuracy for predicting response by ROC curve analysis of 0.65, with a sensitivity of 55% and a specificity of 75%. They also found a difference regarding histology subtype, showing a significant association between adenocarcinoma OR by RECIST and CEA-response which was only barely significant with the squamous histology. Additionally, they did find a relation between marker response and OS. Jin et al. [34] also assessed the value of CEA in response prediction in advanced NSCLC receiving platinum-based CT. They reported a significant association between the change in CEA and OR, time-to progression and OS; they did not, however, evaluated the possible implications of histologic subtypes. They also did not report the percentage reduction of CEA value nor its sensitivity and specificity.

In our study with advanced NSCLC patients receiving platinum-based CT and TKI therapy (in a small subset of patients), we corroborated the findings of previous investigators on the usefulness of CEA in predicting response to treatment in a prospective fashion and, to our knowledge, this is the largest cohort of patients in which the role of CEA as a predictive tool is validated. Using a set point of ≥14% reduction of CEA levels after two cycles of treatment, we managed to increase the accuracy for response prediction by ROC curve analysis (AURC, 0.94), and to increase the sensitivity and specificity of the marker for OR prediction. Likewise, we found that an increase of CEA values after two cycles ≥18% also correlated well with PD with a sensitivity of 85%. Additionally, when CEA changes were evaluated according to histology, not only adenocarcinoma patients showed a difference in tumor response, but these changes were also noted in non-adenocarcinoma patients.

We described a significant association between the reduction of CEA and PFS but not OS, interestingly, OR by standard RECIST technique also did not show significance for OS. We postulate that the reason that the CEA reduction was not associated with a prolonged OS is that in this cohort of patients, most of them received further treatment with 2 to 4 lines of chemotherapy and/or tyrosine kinase inhibitors.

Perhaps the better performance of CEA in our study is due to the high representation of patients with adenocarcinoma (84%) in a large cohort. It has previously been demonstrated that CEA levels correlate more accurately to prediction of prognosis and OR in adenocarcinoma histology than non-adenocarcinoma [15,35]. Matsuoka et al. [7] showed a relationship between histologic subtype and the usefulness of CEA as a prognostic indicator in NSCLC patients with pathologic stage I, showing that a high preoperative CEA level was associated to shorter disease-free survival and lower 5-year survival rate in adenocarcinoma compared to the squamous histology in whom it was not predictive of survival nor recurrence. Furthermore, a report by Reinmuth et al. [27] evaluating the prognostic impact of CEA in resectable NSCLC patients failed to reach statistical significance, importantly, they had an overrepresentation of patients with squamous-cell carcinoma (46% overall).

In this study, we confirmed that CEA measurements during follow-up are particularly helpful in patients with elevated CEA at diagnosis with measurable disease, although they might be potentially useful for unmeasurable disease as well, such as pleural effusions, diffuse nodules, or tumors with poorly defined margins, according to a previously published study [30].

The usefulness of other tumor markers than CEA has been evaluated in resectable and advanced NSCLC. Of note, the immunometric assay of cytokeratin-19 fragments (CYFRA 21–1) has been the most studied yielding great results as a prognostic [36,37] and OR-predictive tool [15,34,35]. We chose to use only CEA because of its wider availability for routine clinical use and standardized performance.

## Conclusions

A ≥14% reduction of serum CEA level from baseline after 2 cycles of treatment in advanced NSCLC is an accurate measurement of OR compared to RECIST, it has outstanding sensitivity and specificity, and correlates well with PFS especially in adenocarcinoma histology. Contrariwise, an increase of ≥18% of serum CEA levels from baseline is also an accurate measurement of PD. Together with the two previous studies, we demonstrated the predictive value of measuring CEA levels in NSCLC and we propose it to be part of the routine follow-up of advanced NSCLC patients who have increase levels of CEA (>10 mg/dl) at baseline and are receiving platinum-based CT.

### Clinical practice points

•Declining CEA levels have been studied in advanced NSCLC patients with similar results [15,30-32], although, with a smaller cohort of patients and a significant underrepresentation of adenocarcinoma histology [27] in which CEA measurement is most useful.

•In the surgical setting, Matsuoka et al. [33] proved that CEA declining levels are valuable as a prognostic marker of recurrence, also in the adenocarcinoma subtype.

•A decrease of ≥14% of serum CEA level from baseline had 90% specificity for overall tumor response making it a promising tool and a more objective method of evaluating response to chemotherapy in advanced NSCLC patients.

## Abbreviations

CEA: Carcinoembrionic antigen; NSCLC: Non-small cell lung cancer; ROC: Receiver operating characteristics; AURC: Area under the ROC curve; AUC: Area under the curve; OR: Overall response; PFS: Progression free survival; OS: Overall survival; FDG: Fluorodeoxyglucose; PET: Positron emission tomography; PSA: Prostate-specific antigen; ECOG: Eastern Cooperative Oncology Group; TKI: Tyrosine kinase inhibitor; SD: Stable disease; PD: Progressive disease; CI: Confidence interval.

## Competing interests

The authors declare that they have no competing interests.

## Authors’ contributions

OA Conception and design, Financial Suport, provision of study materials or patients, data analisis and interpretation, manuscript writing, final approval of the manuscript. LMB provision of study material or patients, final approval of the manuscript. CVG data analisis and interpretation, manuscript writing, final approval of the manuscript. MM provision of study materials or patients, manuscript writing, final approval of the manuscript. DG data analisis and interpretation, manuscript writing, final approval of the manuscript. OPC data analisis and interpretation, manuscript writing, final approval of the manuscript. EOM provision of study materials or patients, manuscript writing, final approval of the manuscript. SCR manuscript writing, final approval of the manuscript. JAA manuscript writing, final approval of the manuscript. All authors read and approved the final manuscript.

## Pre-publication history

The pre-publication history for this paper can be accessed here:

http://www.biomedcentral.com/1471-2407/13/254/prepub
